# ﻿Pholcid spiders of the *Pholcusphungiformes* species-group (Araneae, Pholcidae) from Liaoning Province, China: an overview, with description of a new species

**DOI:** 10.3897/zookeys.1156.98331

**Published:** 2023-03-24

**Authors:** Fangyu Zhao, Tian Jiang, Lan Yang, Qiaoqiao He, Guo Zheng, Zhiyuan Yao

**Affiliations:** 1 College of Life Science, Shenyang Normal University, Shenyang 110034, Liaoning, China Shenyang Normal University Shenyang China; 2 Liaoning Key Laboratory of Evolution and Biodiversity, Shenyang 110034, Liaoning, China Liaoning Key Laboratory of Evolution and Biodiversity Shenyang China; 3 Liaoning Key Laboratory for Biological Evolution and Agricultural Ecology, Shenyang 110034, Liaoning, China Liaoning Key Laboratory for Biological Evolution and Agricultural Ecology Shenyang China

**Keywords:** Biodiversity, daddy-long-legs spider, morphology, Northeast Asia, taxonomy

## Abstract

Species of the *Pholcusphungiformes* group exhibit high diversity in Liaoning Province of northeastern China. This paper summarizes the current knowledge on this species-group from this area. A checklist of 22 species recorded from this province is given, accompanied with a distribution map of the species. *Pholcusxiuyan* Zhao, Zheng & Yao, **sp. nov.** (♂♀) is described as new to science, and *P.yuhuangshan* Yao & Li, 2021 is reported from Liaoning for the first time.

## ﻿Introduction

The spider family Pholcidae C.L. Koch, 1850 currently contains 97 genera and 1896 species (World Spider Catalog 2022) classified within five subfamilies: Arteminae Simon, 1893, Modisiminae Simon, 1893, Ninetinae Simon, 1890, Pholcinae C.L. Koch, 1850, and Smeringopinae Simon, 1893 (Huber 2011a; Dimitrov et al. 2013; Eberle et al. 2018). *Pholcus* Walckenaer, 1805 is the most diverse genus of the family, comprising 375 described species (World Spider Catalog 2022). These species belong to 21 species-groups, of which the *phungiformes* group is highly diverse and contains 94 species (Huber 2011b; Wang et al. 2020; Yao et al. 2021; Lu et al. 2022). This species-group is mainly distributed in northeastern China and the Korean Peninsula. Liaoning is a province in northeastern China and lies northwest of North Korea. The exploration of pholcid spiders from Liaoning was started rather recently, especially for the *phungiformes* species-group. Song and Ren (1994) were the first authors to record pholcids from Liaoning, and they described two species, *P.guani* Song & Ren, 1994 from Beizhen County and *P.gaoi* Song & Ren, 1994 from Kuandian County; the latter species belongs to the *phungiformes* species-group. Song et al. (1999) described the second species of this species-group, *P.suizhongicus* Zhu & Song, 1999, from Suizhong County. Nearly 10 years later, Zhang and Zhu (2009) described two species from Fenghuangshan Mountain, namely *P.fengcheng* Zhang & Zhu, 2009 and *P.phoenixus* Zhang & Zhu, 2009. During the following decade (2010–2019), only eight species of this group have been described from Liaoning (Tong and Ji 2010; Yao and Li 2012; Peng and Zhang 2013; Liu and Tong 2015). Wang et al. (2020) estimated that a large part of the *phungiformes* species-group diversity likely remains undiscovered in the Changbai Mountains in northeastern China, especially in Liaoning. For this reason, a one-month-long expedition to the Changbai Mountains was undertaken, which resulted in 11 new species being reported, including eight from Liaoning (Yao et al. 2021). To date, 20 species of this species-group have been recorded from Liaoning, most of which were collected from rock walls or at cave entrances.

**Figure 1.**
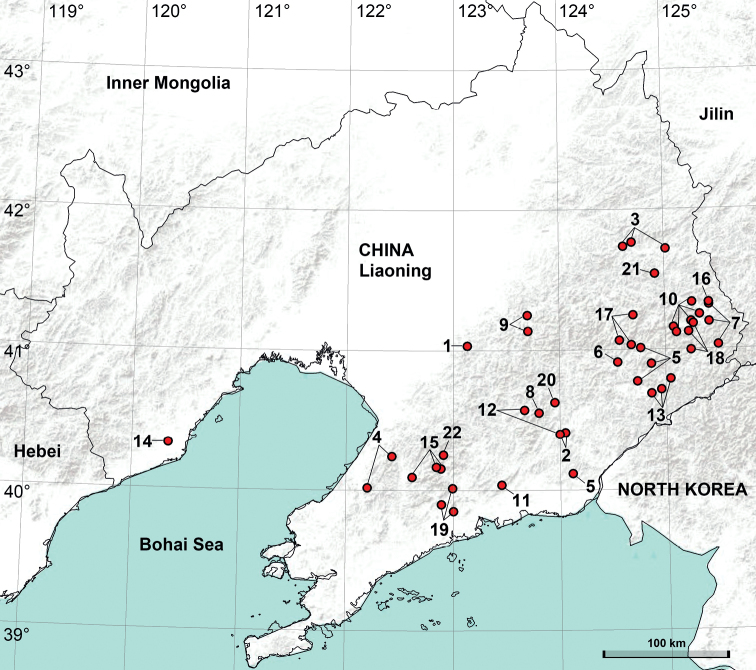
Distribution of the *Pholcusphungiformes* species-group in Liaoning, China: 1 = *P.decorus*, 2 = *P.fengcheng*, 3 = *P.foliaceus*, 4 = *P.gaizhou*, 5 = *P.gaoi*, 6 = *P.guanshui*, 7 = *P.hamatus*, 8 = *P.jiguanshan*, 9 = *P.jiuwei*, 10 = *P.longxigu*, 11 = *P.luoquanbei*, 12 = *P.phoenixus*, 13 = *P.shenshi*, 14 = *P.suizhongicus*, 15 = *P.tianmenshan*, 16 = *P.tongi*, 17 = *P.wangi*, 18 = *P.wangtian*, 19 = *P.xianrendong*, 20 = *P.yaoshan*, 21 = *P.yuhuangshan*, 22 = *P.xiuyan* sp. nov.

**Figure 2.**
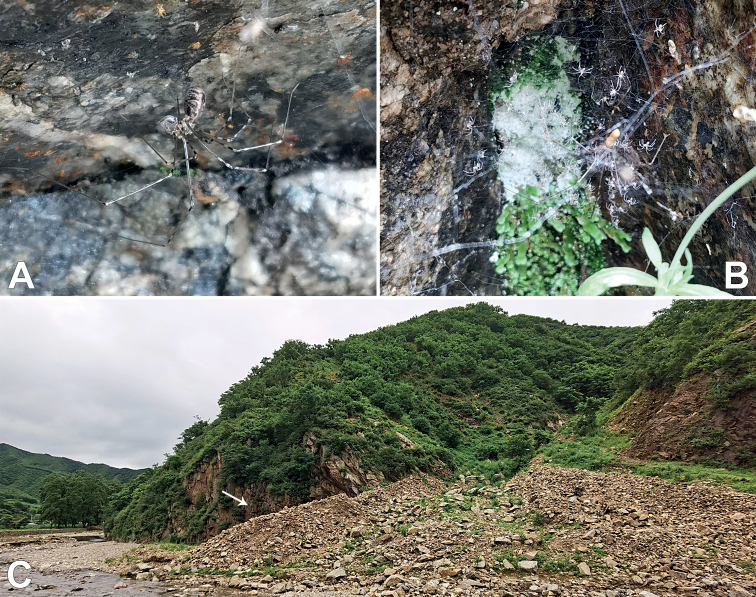
*Pholcusxiuyan* sp. nov., living specimens and habitat **A, B** females and juveniles on rock walls **C** habitat, arrow indicates collecting site.

The present study provides a checklist of the *phungiformes* species-group from Liaoning and a distribution map of all of the species (Figs 1, 2). This study also describes a species new to science and reports a species from this region for the first time.

## ﻿Materials and methods

Specimens were examined and measured with a Leica M205 C stereomicroscope. The left male palp was photographed. The epigyne was photographed before dissection. The vulva was treated in a 10% warm solution of potassium hydroxide (KOH) to dissolve soft tissues before illustration. Images were captured with a Canon EOS 750D wide zoom digital camera (24.2 megapixels) mounted on the stereomicroscope mentioned above and assembled using Helicon Focus v. 3.10.3 image stacking software (Khmelik et al. 2005). All measurements are given in millimeters (mm). Leg measurements are shown as: total length (femur, patella, tibia, metatarsus, tarsus). Leg segments were measured on their dorsal side. The distribution map was generated with ArcGIS v. 10.2 (ESRI Inc.). The specimens studied are preserved in 75% ethanol and deposited in the College of Life Science, Shenyang Normal University (**SYNU**) in Liaoning, China.

Terminology and taxonomic descriptions follow Huber (2011b) and Yao et al. (2015, 2021). The following abbreviations are used in the descriptions: **ALE** = anterior lateral eye, **AME** = anterior median eye, **PME** = posterior median eye, **L/d** = length/diameter ratio; used in the illustrations: **b** = bulb, **da** = distal apophysis, **e** = embolus, **fa** = frontal apophysis, **pa** = proximo-lateral apophysis, **pp** = pore plate, **pr** = procursus, **u** = uncus.

## ﻿Taxonomic accounts

**Family Pholcidae C.L. Koch, 1850**

**Subfamily Pholcinae C.L. Koch, 1850**

### ﻿Genus Pholcus

Taxon classificationAnimaliaAraneaePholcidae

Walckenaer, 1805

B41A17A8-CBA7-5D27-B4E0-466524EE0A99

#### Type species.

*Araneaphalangioides* Fuesslin, 1775.

***Pholcusphungiformes* species-group from Liaoning Province**

**Diagnosis and description.** See Huber (2011b) and Yao et al. (2021).

### ﻿1. Pholcus decorus

Taxon classificationAnimaliaAraneaePholcidae

Yao & Li, 2012

B39E89DE-FEAE-5DB8-9EA4-5F46E07960BC

Pholcus decorus Yao & Li, 2012: 14, figs 55A–D, 56A–E, 57A–E, 58A–D (♂♀).
Pholcus decorus Yao et al., 2021: S6, fig. 2B.1 (♂).

#### Distribution.

China (Anshan in Liaoning; habitat: rock walls).

### ﻿2. Pholcus fengcheng

Taxon classificationAnimaliaAraneaePholcidae

Zhang & Zhu, 2009

2FFB0AAC-F18E-5E10-B222-9CB437D03017

Pholcus fengcheng Zhang & Zhu, 2009: 28, fig. 11A–I (♂♀).
Pholcus fengcheng Yao & Li, 2012: 16, figs 63A–D, 64A–C (♂♀). Yao et al. 2021: S7, fig. 2B.2 (♂).

#### New material examined.

1♂ (SYNU-Ar00001F), roadside of G304 (40°24.667'N, 124°3.067'E, 139 m), near Fenghuangshan Mountain, Fengcheng, Dandong, **Liaoning**, **China**, 13 July 2020, Z Yao leg.

#### Distribution.

China (Fengcheng in Liaoning; habitat: rock walls).

### ﻿3. Pholcus foliaceus

Taxon classificationAnimaliaAraneaePholcidae

Peng & Zhang, 2013

CA4A750D-7179-5DDF-A0ED-2151CE74B7A4

Pholcus foliaceus Peng & Zhang, 2013: 75, figs 1A–G, 2A–F (♂♀).
Pholcus foliaceus Yao et al., 2021: S7, figs 2B.3, S4A–D (♂♀).

#### New material examined.

2♂ (SYNU-Ar00002F, Ar00003F) and 2♀ (SYNU-Ar00004F, Ar00005F), roadside of S202 (41°44.117'N, 124°36.867'E, 119 m), near Houshi National Forest Park, Muqi Town, Xinbin County, Fushun, **Liaoning**, **China**, 23 June 2020, Z Yao leg.

#### Distribution.

China (Qingyuan County and Xinbin County in Liaoning; habitat: rock walls).

### ﻿4. Pholcus gaizhou

Taxon classificationAnimaliaAraneaePholcidae

Yao & Li, 2021

5F460A7B-5F40-5A74-8650-CC60E287AECC

Pholcus gaizhou Yao & Li in Yao et al. 2021: S8, figs 2B.4, S5A–D, S6A–H (♂♀).

#### New material examined.

2♂ (SYNU-Ar00006F, Ar00007F) and 3♀ (SYNU-Ar00008F–Ar00010F), roadside (40°1.167'N, 122°12.050'E, 257 m), near Xuemaoshan Scenic Spot, Jiuzhai Town, Gaizhou, Yingkou, **Liaoning**, **China**, 16 July 2020, Z Yao leg.

#### Distribution.

China (Gaizhou in Liaoning; habitat: rock walls).

### ﻿5. Pholcus gaoi

Taxon classificationAnimaliaAraneaePholcidae

Song & Ren, 1994

9CF848CC-03EF-53E7-93D4-884CF97F2893

Pholcus gaoi Song & Ren, 1994: 20, figs 1–7 (♂♀).
Pholcus gaoi Song et al., 1999: 57, fig. 23L–O (♂♀). Zhang and Zhu 2009: 29, fig. 12A–G (♂♀). Yao and Li 2012: 17, figs 69A–D, 70A–C (♂♀). Yao et al. 2021: S9, fig. 2B.5 (♂).

#### New material examined.

2♂ (SYNU-Ar00011F, Ar00012F) and 1♀ (SYNU-Ar00013F), roadside of Sandaogoumen (40°53.950'N, 124°51.900'E, 431 m), Xinfeng Village, Dachuantou Town, Kuandian County, Dandong, **Liaoning**, **China**, 8 July 2020, Z Yao leg. 3♂ (SYNU-Ar00014F–Ar00016F) and 1♀ (SYNU-Ar00017F), roadside of X627 (41°0.633'N, 124°45.950'E, 520 m), Jiangjunling, Bahechuan Town, Kuandian County, Dandong, **Liaoning**, **China**, 8 July 2020, Z Yao leg.

#### Distribution.

China (Kuandian County in Liaoning; habitat: rock walls).

### ﻿6. Pholcus guanshui

Taxon classificationAnimaliaAraneaePholcidae

Yao & Li, 2021

7554BDA0-A01E-5126-9578-2575E73A524F

Pholcus guanshui Yao & Li in Yao et al. 2021: S10, figs 2B.6, S7A–D, S8A–H (♂♀).

#### Distribution.

China (Kuandian County in Liaoning; habitat: rock walls).

### ﻿7. Pholcus hamatus

Taxon classificationAnimaliaAraneaePholcidae

Tong & Ji, 2010

B7B27F76-6E40-5058-AD13-04CF0EAF3A32

Pholcus hamatus Tong & Ji, 2010: 98, figs 1a–c, j, 2a–g (♂♀).
Pholcus hamatus Yao et al., 2021: S11, figs 2B.7, S9A–D (♂♀).

#### New material examined.

1♂ (SYNU-Ar00018F) and 3♀ (SYNU-Ar00019F–Ar00021F), roadside of G506 (41°11.733'N, 125°25.100'E, 349 m), Huanggouli Village, Yahe Town, Huanren County, Benxi, **Liaoning**, **China**, 27 June 2020, Z Yao leg. 2♂ (SYNU-Ar00022F, Ar00023F) and 3♀(SYNU-Ar00024F–Ar00026F), roadside of G506 (41°1.783'N, 125°30.000'E, 179 m), Dawudaoyangcha, Gangouzi Village, Shajianzi Town, Huanren County, Benxi, **Liaoning**, **China**, 27 June 2020, Z Yao leg.

#### Distribution.

China (Huanren County in Liaoning; habitat: rock walls).

### ﻿8. Pholcus jiguanshan

Taxon classificationAnimaliaAraneaePholcidae

Yao & Li, 2021

8FE82148-E4B9-5964-8CAF-8A50C93D1A04

Pholcus jiguanshan Yao & Li in Yao et al. 2021: S12, figs 2B.8, S10A–D, S11A–H (♂♀).

#### Distribution.

China (Fengcheng in Liaoning; habitat: rock walls).

### ﻿9. Pholcus jiuwei

Taxon classificationAnimaliaAraneaePholcidae

Tong & Ji, 2010

4D5E4BE4-F043-569D-9BA8-27105C14F6B6

Pholcus jiuwei Tong & Ji, 2010: 99, figs 1d–f, k, 3a–g (♂♀).
Pholcus jiuwei Yao et al., 2021: S13, figs 2B.9, S12A–D (♂♀).

#### New material examined.

2♂ (SYNU-Ar00027F, Ar00028F) and 2♀ (SYNU-Ar00029F, Ar00030F), roadside (41°8.233'N, 125°42.217'E, 227 m), near Benxi Grand Canyon, Nanfen District, Benxi, **Liaoning**, **China**, 12 July 2020, Z Yao leg.

#### Distribution.

China (Benxi and Pingshan County in Liaoning; habitat: rock walls).

### ﻿10. Pholcus longxigu

Taxon classificationAnimaliaAraneaePholcidae

Yao & Li, 2021

63BF9EFE-C684-58CF-A373-888BDA2C0006

Pholcus longxigu Yao & Li in Yao et al. 2021: S14, figs 2B.11, S13A–D, S14A–H (♂♀).

#### New material examined.

1♂ (SYNU-Ar00031F) and 3♀ (SYNU-Ar00032F–Ar00034F), roadside of S201 (41°20.117'N, 125°15.400'E, 291 m), Wudaohezi Village, Huanren Town, Huanren County, Benxi, **Liaoning**, **China**, 24 June 2020, Z Yao leg. 1♂ (SYNU-Ar00035F), roadside of G201 (41°14.867'N, 125°19.650'E, 294 m), Shihada Village, Yahe Town, Huanren County, Benxi, **Liaoning**, **China**, 26 June 2020, Z Yao leg. 1♂ (SYNU-Ar00036F) and 1♀ (SYNU-Ar00037F), roadside of G201 (41°11.967'N, 125°14.817'E, 276 m), near Lianhe Bridge, Lianhe Village, Yahe Town, Huanren County, Benxi, **Liaoning**, **China**, 26 June 2020, Z Yao leg. 2♂ (SYNU-Ar00038F, Ar00039F) and 1♀ (SYNU-Ar00040F), roadside of G201 (41°6.467'N, 125°7.217'E, 305 m), near Daqinggou Village, Pulepu Town, Benxi, **Liaoning**, **China**, 6 July 2020, Z Yao leg.

#### Distribution.

China (Benxi and Huanren County in Liaoning; habitat: rock walls).

### ﻿11. Pholcus luoquanbei

Taxon classificationAnimaliaAraneaePholcidae

Yao & Li, 2021

6C5142B4-78FC-53B1-91DC-9BB023463AC0

Pholcus luoquanbei Yao & Li in Yao et al. 2021: S15, figs 2B.12, S15A–D, S16A–H (♂♀).

#### Distribution.

China (Xiuyan County in Liaoning; habitat: rock walls).

### ﻿12. Pholcus phoenixus

Taxon classificationAnimaliaAraneaePholcidae

Zhang & Zhu, 2009

97BA9834-ECE1-531F-A579-DAF879C62403

Pholcus phoenixus Zhang & Zhu, 2009: 69, figs 37A–I, 38A–I (♂♀).
Pholcus phoenixus Yao & Li, 2012: 30, figs 144A–D, 145A–C (♂♀). Yao et al. 2021: S17, fig. 2B.14 (♂).

#### New material examined.

3♂ (SYNU-Ar00041F–Ar00043F) and 2♀ (SYNU-Ar00044F, Ar00045F), roadside of Maqing Road (40°34.483'N, 123°40.050'E, 243 m), near Qingliangshan Scenic Spot, Qingliangshan Village, Qinglingshan Town, Xiuyan County, Anshan, **Liaoning**, **China**, 13 July 2020, Z Yao leg.

#### Distribution.

China (Fengcheng and Xiuyan County in Liaoning; habitat: rock walls).

### ﻿13. Pholcus shenshi

Taxon classificationAnimaliaAraneaePholcidae

Yao & Li, 2021

B3B91A96-DA8D-5A48-9685-1283C9573C6B

Pholcus shenshi Yao & Li in Yao et al. 2021: S18, figs 2B.15, S17A–D, S18A–H (♂♀).

#### New material examined.

1♂ (SYNU-Ar00046F) and 2♀ (SYNU-Ar00047F, Ar00048F), roadside of Kuanbei Road (40°42.883'N, 124°57.483'E, 315 m), Shanghaozigou Village, Hongshi Town, Kuandian County, Dandong, **Liaoning**, **China**, 7 July 2020, Z Yao leg. 1♂ (SYNU-Ar00049F) and 1♀ (SYNU-Ar00050F), roadside of Kuanbei Road (40°41.217'N, 124°51.867'E, 439 m), Shangchangyinzi Village, Shihugou Town, Kuandian County, Dandong, **Liaoning**, **China**, 7 July 2020, Z Yao leg.

#### Distribution.

China (Kuandian County in Liaoning; habitat: rock walls).

### ﻿14. Pholcus suizhongicus

Taxon classificationAnimaliaAraneaePholcidae

Zhu & Song, 1999

1D444285-C8FC-5FC3-BBAB-01967E23B404

Pholcus suizhongicus Zhu & Song in Song et al. 1999: 59, fig. 25A–H (♂♀).
Pholcus suizhongicus Zhang & Zhu, 2009: 89, fig. 51A–H (♂♀). Yao and Li 2012: 33, figs 167A–D, 168A–C (♂♀).

#### Distribution.

China (Suizhong County in Liaoning; habitat: unknown).

### ﻿15. Pholcus tianmenshan

Taxon classificationAnimaliaAraneaePholcidae

Yao & Li, 2021

26B39A30-A16A-5351-95C6-64190ECACCAF

Pholcus tianmenshan Yao & Li in Yao et al. 2021: S19, figs 2B.17, S19A–D, S20A–H (♂♀).

#### New material examined.

2♂ (SYNU-Ar00051F, Ar00052F) and 2♀ (SYNU-Ar00053F, Ar00054F), roadside of Beitu Road (40°9.983'N, 122°50.567'E, 345 m), Taipingzhuang Village, Kuangdonggou Town, Gaizhou, Yingkou, **Liaoning**, **China**, 15 July 2020, Z Yao leg. 1♂ (SYNU-Ar00055F) and 3♀ (SYNU-Ar00056F–Ar00058F), roadside (40°5.617'N, 122°37.050'E, 227 m), near Chishan Scenic Spot, Wanfu Town, Gaizhou, Yingkou, **Liaoning**, **China**, 15 July 2020, Z Yao leg.

#### Distribution.

China (Gaizhou and Zhuanghe in Liaoning; habitat: rock walls).

### ﻿16. Pholcus tongi

Taxon classificationAnimaliaAraneaePholcidae

Yao & Li, 2012

0856615B-0A35-5C51-A44F-0A5C31946F74

Pholcus tongi Yao & Li, 2012: 34, figs 173A–D, 174A–E, 175A–D, 176A–D (♂♀).
Pholcus tongi Yao et al., 2021: S20, fig. 2B.18 (♂).

#### Distribution.

China (Huanren County in Liaoning; habitat: rock walls).

### ﻿17. Pholcus wangi

Taxon classificationAnimaliaAraneaePholcidae

Yao & Li, 2012

9B5E83E7-6EB2-5ECB-8F51-D43BF7904183

Pholcus wangi Yao & Li, 2012: 37, figs 191A–D, 192A–E, 193A–D, 194A–D (♂♀).
Pholcus wangi Yao et al., 2021: S21, fig. 2B.19 (♂).

#### New material examined.

1♂ (SYNU-Ar00059F) and 2♀ (SYNU-Ar00060F, Ar00061F), roadside of S309 (41°2.017'N, 124°40.667'E, 306 m), Liming Village, Shuangshanzi Town, Kuandian County, Dandong, **Liaoning**, **China**, 10 July 2020, Z Yao leg. 3♂ (SYNU-Ar00062F–Ar00064F) and 3♀ (SYNU-Ar00065F–Ar00067F), roadside of G506 (41°14.783'N, 124°42.000'E, 628 m), Dongyingfang Town, Benxi County, Benxi, **Liaoning**, **China**, 10 July 2020, Z Yao leg.

#### Distribution.

China (Benxi County and Kuandian County in Liaoning; habitat: rock walls).

### ﻿18. Pholcus wangtian

Taxon classificationAnimaliaAraneaePholcidae

Tong & Ji, 2010

B4AEC971-3477-55AA-AD2D-29FE27C2EFE5

Pholcus wangtian Tong & Ji, 2010: 102, figs 1g–i, l, 4a–f (♂♀).
Pholcus wangtian Yao et al., 2021: S23, figs 2B.21, S23A–D (♂♀).

#### New material examined.

1♂ (SYNU-Ar00068F) and 2♀ (SYNU-Ar00069F, Ar00070F), Fenglingu Forest Park (41°7.433'N, 125°13.400'E, 392 m), Xiangyang Town, Huanren County, Benxi, **Liaoning**, **China**, 26 June 2020, Z Yao leg. 2♂ (SYNU-Ar00071F, Ar00072F) and 2♀ (SYNU-Ar00073F, Ar00074F), roadside (40°59.683'N, 125°14.533'E, 212 m), near Manjiazhai, Qingshangou Village, Qingshangou Town, Kuandian County, Dandong, **Liaoning**, **China**, 7 July 2020, Z Yao leg.

#### Distribution.

China (Huanren County and Kuandian County in Liaoning; habitat: rock walls and a cave entrance).

### ﻿19. Pholcus xianrendong

Taxon classificationAnimaliaAraneaePholcidae

Liu & Tong, 2015

6A9F021E-A26B-56EE-9E3A-5D08E1301A59

Pholcus xianrendong Liu & Tong, 2015: 32, figs 1A–J, 2A–F (♂♀).
Pholcus xianrendong Yao et al., 2021: S24, figs 2B.22, S24A–D (♂♀).

#### New material examined.

3♂ (SYNU-Ar00075F–Ar00077F) and 2♀ (SYNU-Ar00078F, Ar00079F), roadside of S203 (39°50.983'N, 123°0.150'E, 96 m), Dawangtun, Sijia Village, Daying Town, Zhuanghe, Dalian, **Liaoning**, **China**, 15 July 2020, Z Yao leg.

#### Distribution.

China (Zhuanghe in Liaoning; habitat: rock walls).

### ﻿20. Pholcus yaoshan

Taxon classificationAnimaliaAraneaePholcidae

Yao & Li, 2021

AF5C617A-D726-5F9A-A6FF-632A8C2A1F81

Pholcus yaoshan Yao & Li in Yao et al. 2021: S25, figs 2B.24, S27A–D, S28A–H (♂♀).

#### Distribution.

China (Xiuyan County in Liaoning; habitat: rock walls).

### ﻿21. Pholcus yuhuangshan

Taxon classificationAnimaliaAraneaePholcidae

Yao & Li, 2021

F4F1B6A6-D6C3-5680-8551-1ED9D3211F6F

Pholcus yuhuangshan Yao & Li in Yao et al. 2021: S27, figs 2B.25, S1A, S29A–D, S30A–H (♂♀).

#### New material examined.

2♂ (SYNU-Ar00080F, Ar00081F) and 1♀ (SYNU-Ar00082F), roadside of S201 (41°32.400'N, 124°54.717'E, 118 m), Chaluzi Village, Yüshu Town, Xinbin County, Fushun, **Liaoning**, **China**, 24 June 2020, Z Yao leg. (New record for Liaoning)

#### Distribution.

China (Xinbin County in Liaoning; habitat: rock walls).

### ﻿22. Pholcus xiuyan

Taxon classificationAnimaliaAraneaePholcidae

Zhao, Zheng & Yao sp. nov.

BE162775-6FE9-5DB6-8E27-154FF7C22D7A

https://zoobank.org/5583C31D-4B7E-4C42-8A34-20BB8663778F

Figs 3 , 4

#### Remarks.

**Figure 3.**
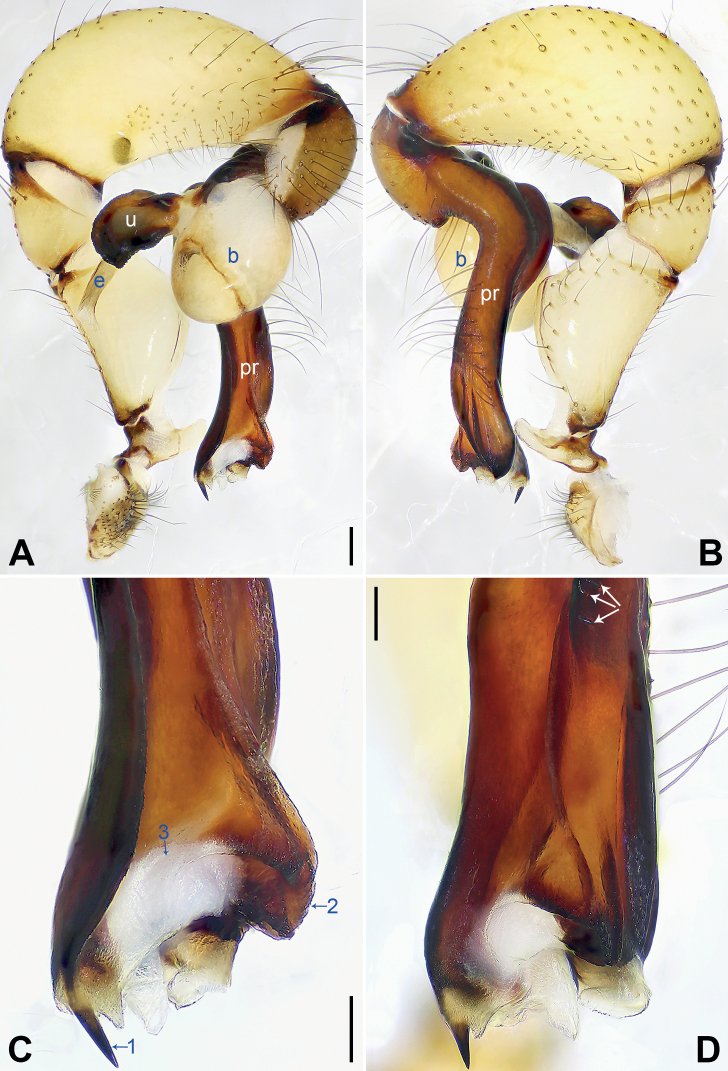
*Pholcusxiuyan* sp. nov., holotype male **A, B** palp (**A** prolateral view **B** retrolateral view) **C, D** distal part of procursus (**C** prolateral view, arrow 1 indicates spine-shaped distal apophysis, arrow 2 indicates sclerotized dorsal protrusion, arrow 3 indicates subdistal membranous process **D** dorsal view, arrows indicate dorsal spines). Abbreviations: b = bulb, e = embolus, pr = procursus, u = uncus. Scale bars: 0.20 mm (**A, B**); 0.10 mm (**C, D**).

**Figure 4.**
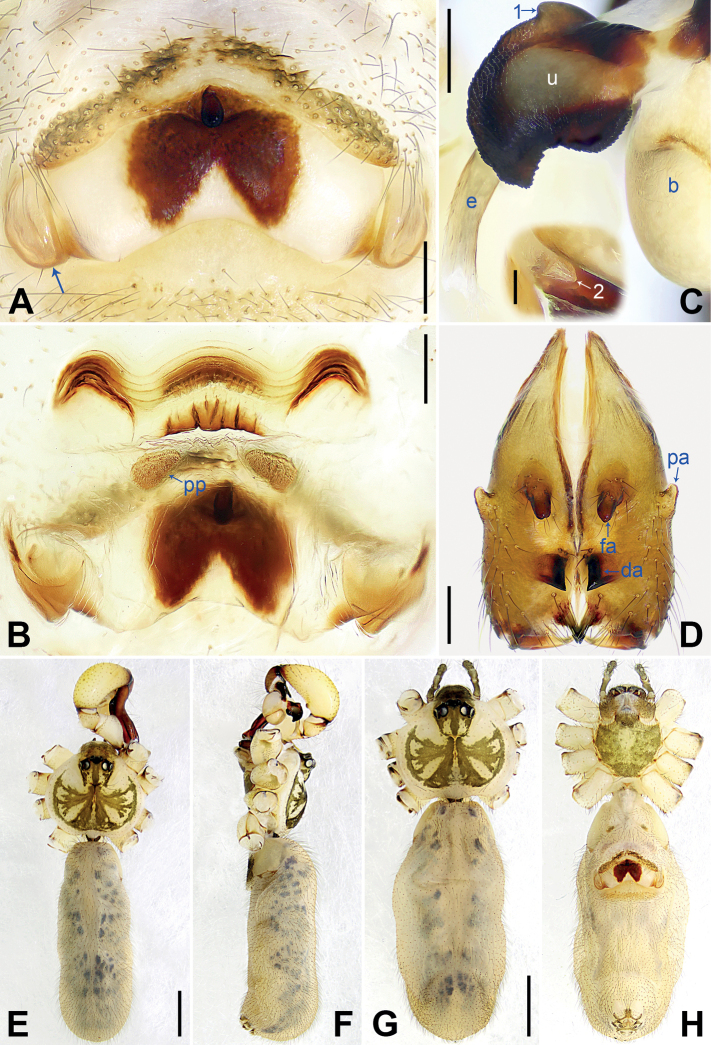
*Pholcusxiuyan* sp. nov., holotype male (**C–F**) and paratype female (**A, B, G, H**) **A** epigyne, ventral view, arrow indicates lateral protrusion **B** vulva, dorsal view **C** bulbal apophyses, prolateral view (the insert is retrolateral view of “pseudo-appendix”), arrow 1 indicates semicircular proximal apophysis, arrow 2 indicates “pseudo-appendix” **D** chelicerae, frontal view **E–H** habitus (**E, G** dorsal view **F** lateral view **H** ventral view). Abbreviations: b = bulb, da = distal apophysis, e = embolus, fa = frontal apophysis, pa = proximo-lateral apophysis, pp = pore plate, u = uncus. Scale bars: 0.20 mm (**A–D**); 0.05 mm (the insert in **C**); 1.00 mm (**E–H**).

This new species is assigned to the *phungiformes* group by the following combination of characters: the male chelicerae with frontal apophyses (arrow fa in Fig. 4D), the male palpal tibia with a prolatero-ventral projection (Fig. 3A), the procursus with dorsal spines (arrows in Fig. 3D), the uncus with a “pseudo-appendix” (arrow 2 in Fig. 4C), and the epigyne with a knob (Fig. 4A).

#### Type material.

***Holotype***: ♂ (SYNU-Ar00251), Jiaxigou (40°15.200'N, 122°54.567'E, 318 m), Taipingling Village, Shihuiyao Town, Xiuyan County, Anshan, **Liaoning**, **China**, 13 July 2022, G Zheng, L Xiang & N Li leg. ***Paratypes***: 1♂ (SYNU-Ar00252) and 2♀ (SYNU-Ar00253, Ar00254), same data as for the holotype.

#### Etymology.

The specific name refers to the type locality; noun in apposition.

#### Diagnosis.

The new species resembles *P.brevis* Yao & Li, 2012 (Yao and Li 2012: 12, figs 39A–D, 40A–E, 41A–D, 42A–D) with similar bulbal apophyses (Fig. 4C) and epigynal plate (Fig. 4A), but it can be easily distinguished by the procursus with a sclerotized dorsal protrusion (arrow 2 in Fig. 3C; a flat dorsal sclerite in *P.brevis*), the strong male cheliceral frontal apophyses (arrow fa in Fig. 4D; frontal apophyses indistinct in *P.brevis*), the epigyne with a pair of lateral protrusions (arrow in Fig. 4A; absent in *P.brevis*), the wavy vulval anterior arch (Fig. 4B; slightly curved anterior arch in *P.brevis*), and the male clypeus without frontal apophysis (Fig. 4E; present in *P.brevis*); also distinguished from all of its known congeners in Xiuyan County by the following combination of characters: the procursus with sclerotized, raised prolateral edge bearing a spine-shaped distal apophysis (arrow 1 in Fig. 3C) and a sclerotized dorsal protrusion (arrow 2 in Fig. 3C), the semitransparent “pseudo-appendix” (arrow 2 in Fig. 4C), the strong male cheliceral frontal apophyses (arrow fa in Fig. 4D), the epigyne with a pair of lateral protrusions (arrow in Fig. 4A), and the wavy vulval anterior arch (Fig. 4B).

#### Description.

**Male** (***holotype***, SYNU-Ar00251): total length 6.60 (6.73 with clypeus), prosoma 2.00 long, 2.15 wide, opisthosoma 4.60 long, 1.88 wide. Leg I: 49.82 (12.37, 0.91, 12.63, 20.90, 3.01), leg II: 34.84 (9.62, 0.85, 8.78, 13.65, 1.94), leg III: 19.07 (7.40, 0.73, 6.22, 3.33, 1.39), leg IV: 33.13 (9.62, 0.80, 8.46, 12.69, 1.56); tibia I L/d: 66. Eye interdistances and diameters: PME–PME 0.29, PME 0.17, PME–ALE 0.06, AME–AME 0.08, AME 0.11. Sternum width/length:1.43/1.08. Habitus as in Fig. 4E, F. Dorsal shield of prosoma yellowish, with brown radiating marks and marginal brown bands; ocular area yellowish, with median and lateral brown bands; clypeus and sternum yellowish, with brown marks. Legs overall yellowish, dark brown on patellae and whitish on distal parts of femora and tibiae, with darker rings on subdistal parts of femora and proximal and subdistal parts of tibiae. Opisthosoma yellowish, with dorsal and lateral black spots. Chelicerae (Fig. 4D) with pair of proximo-lateral apophyses, pair of distal apophyses with two teeth each (invisible in frontal view; cf. *P.tianmenshan*, fig. S20D in Yao et al. 2021), and pair of frontal apophyses.

Palp as in Fig. 3A, B; trochanter two times longer than wide, retrolaterally swollen; femur with small retrolatero-proximal protrusion and indistinct ventral protrusion; tibia with prolatero-ventral protrusion; procursus slender, simple proximally but complex distally, with sclerotized, raised prolateral edge bearing spine-shaped distal apophysis (arrow 1 in Fig. 3C), sclerotized dorsal protrusion (arrow 2 in Fig. 3C), subdistal membranous process (arrow 3 in Fig. 3C), and two strong and one slender dorsal spines (arrows in Fig. 3D); uncus curved, with scales and semicircular proximal apophysis (arrow 1 in Fig. 4C); “pseudo-appendix” short and semitransparent (arrow 2 in Fig. 4C); embolus weakly sclerotized, with some transparent distal projections (Fig. 4C). Retrolateral trichobothrium of tibia I at 3% proximally; legs with short vertical setae on tibiae, metatarsi, and tarsi; tarsus I with 38 distinct pseudosegments.

**Female** (***paratype***, SYNU-Ar00253): habitus as in Fig. 4G, H. Total length 5.80 (5.96 with clypeus), prosoma 1.72 long, 2.00 wide, opisthosoma 4.08 long, 1.78 wide; tibia I: 10.05; tibia I L/d: 57. Eye interdistances and diameters: PME–PME 0.22, PME 0.15, PME–ALE 0.04, AME–AME 0.06, AME 0.09. Sternum width/length: 1.22/0.94. Coloration generally as in male, except for dark brown clypeus.

Epigyne (Fig. 4A) postero-medially strongly curved, with median brown marks, short knob and pair of lateral protrusions (arrow in Fig. 4A). Vulva (Fig. 4B) with wavy, medially and laterally sclerotized anterior arch and pair of nearly triangular pore plates.

#### Variation.

Tibia I in paratype male (SYNU-Ar00252): 11.67. Tibia I in another paratype female (SYNU-Ar00254): 9.81.

#### Natural history.

The species was found on rock walls.

#### Distribution.

China (Liaoning, type locality; Fig. 1).

## Supplementary Material

XML Treatment for Pholcus

XML Treatment for Pholcus decorus

XML Treatment for Pholcus fengcheng

XML Treatment for Pholcus foliaceus

XML Treatment for Pholcus gaizhou

XML Treatment for Pholcus gaoi

XML Treatment for Pholcus guanshui

XML Treatment for Pholcus hamatus

XML Treatment for Pholcus jiguanshan

XML Treatment for Pholcus jiuwei

XML Treatment for Pholcus longxigu

XML Treatment for Pholcus luoquanbei

XML Treatment for Pholcus phoenixus

XML Treatment for Pholcus shenshi

XML Treatment for Pholcus suizhongicus

XML Treatment for Pholcus tianmenshan

XML Treatment for Pholcus tongi

XML Treatment for Pholcus wangi

XML Treatment for Pholcus wangtian

XML Treatment for Pholcus xianrendong

XML Treatment for Pholcus yaoshan

XML Treatment for Pholcus yuhuangshan

XML Treatment for Pholcus xiuyan
